# An Audit Cycle Highlighting the Rate of Chlamydia Screening in a Forensic Child and Adolescent Mental Health Unit in Birmingham

**DOI:** 10.1192/bjo.2022.498

**Published:** 2022-06-20

**Authors:** Theresa Ugalahi, Hamid Hassan, John O'Brien

**Affiliations:** 1 Leeds Community Healthcare NHS Trust, Leeds, United Kingdom; 2 Birmingham and Solihull Mental Health Foundation Trust, Birmingham, United Kingdom

## Abstract

**Aims:**

Chlamydia, a sexually transmitted bacterial infection caused by Chlamydia Trachomatis can result in long-term complications for affected individuals. The National chlamydia screening programme recommends screening at-risk young persons, however for the vulnerable patients at the Forensic Child and Adolescent Mental Health Service (FCAMHS), there has been no audit to determine the completion rate. This audit aim to (1) Determine the demographics of young persons on admission (2) To determine the rate of chlamydia screening as well as the percentage of patients who qualified for a Chlamydia screening(3) To determine the rate of documentation for completed tests.

**Methods:**

This was a retrospective study. The medical electronic records of patients who met the inclusion criteria was searched. All the three mixed-sex adolescent forensic wards (2 medium secure units and one low secure unit) at FCAMHS Ardenleigh, Birmingham were sampled.

All patients that were on admission aged above 15 years of age were recruited.

A total sample size of 19 was obtained for the initial audit and 12 for the re-audit.

Data collection

Data were collected by the author for the initial-audit and re-audit by searching the clinical progress notes, the investigation results and the physical health rethink forms. An excel software was used for analysis.

**Results:**

Demographics

There were 11 males (57.9%) and 8 females (42.1%) in the initial audit

In the re-audit, there were 7 males (58.3) and 5 females (41.7). Some of the patients were still on admission at the time of the re-audit, hence the percentages were calculated differently. The mean age and average length of admission was also calculated.

Chlamydia screening

In the initial audit, the percentage of patients tested for Chlamydia was 11.5%, even though 36.8% of patients met the criteria for Chlamydia screening. In the re-audit, 25.0% were tested, and 41.7% met the criteria for Chlamydia screening.

Physical health (Rethink) forms

The physical health form was completed for majority of patients 73.7% in the initial audit although, this was not compatible with screening rates. Before the re-audit was concluded, the physical health forms were no longer in use.

**Conclusion:**

The audit highlighted an overall improvement in the rate of screening following recommendations from initial audit. The inclusion of Chlamydia screening in admission processes could be useful in improving sexual health.

